# Supplementary figures and images for: Assessing the prognostic value of KRAS mutation combined with tumor size in stage I-II non-small cell lung cancer: a retrospective analysis
Source: Front Oncol. 2024 May 31;14:1396285. doi: 10.3389/fonc.2024.1396285 (PMC11176435; doi:10.3389/fonc.2024.1396285)

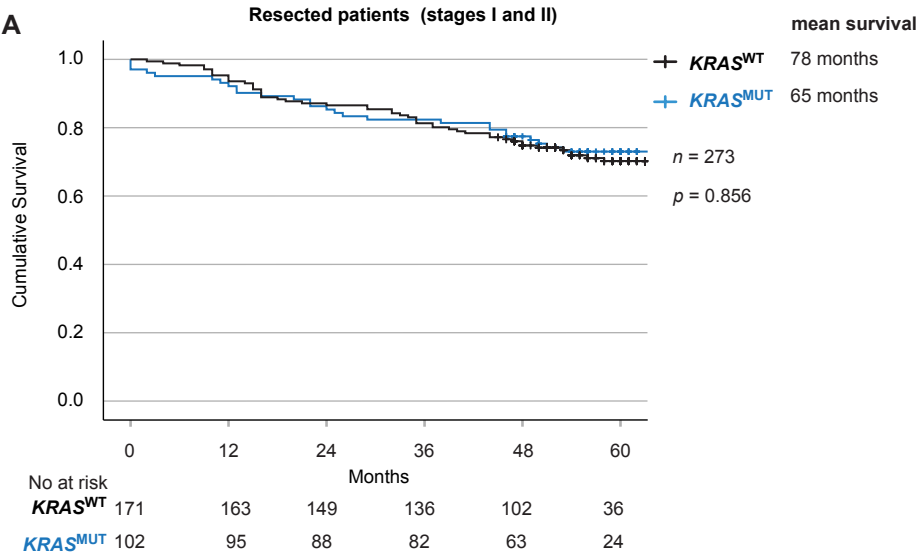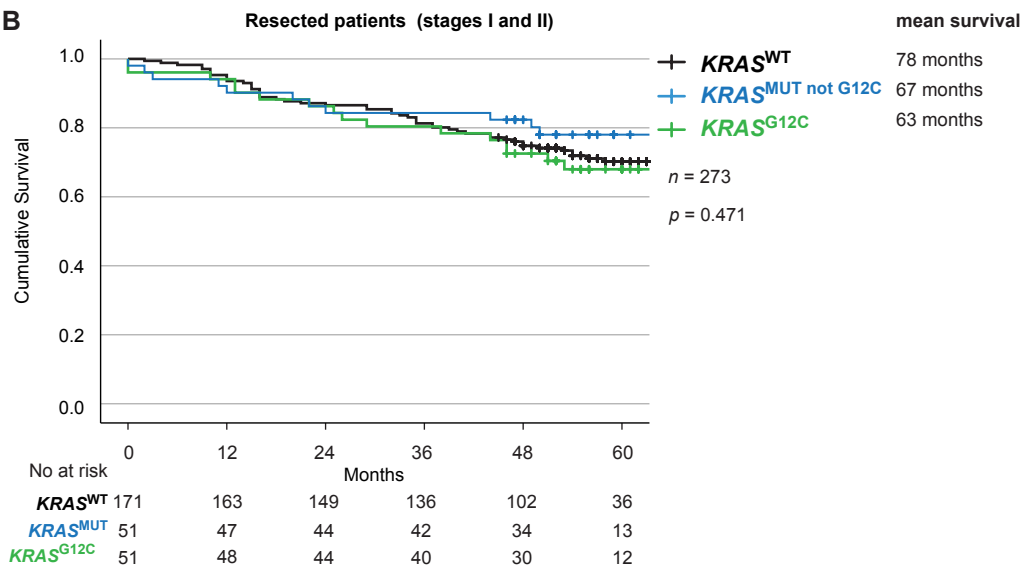

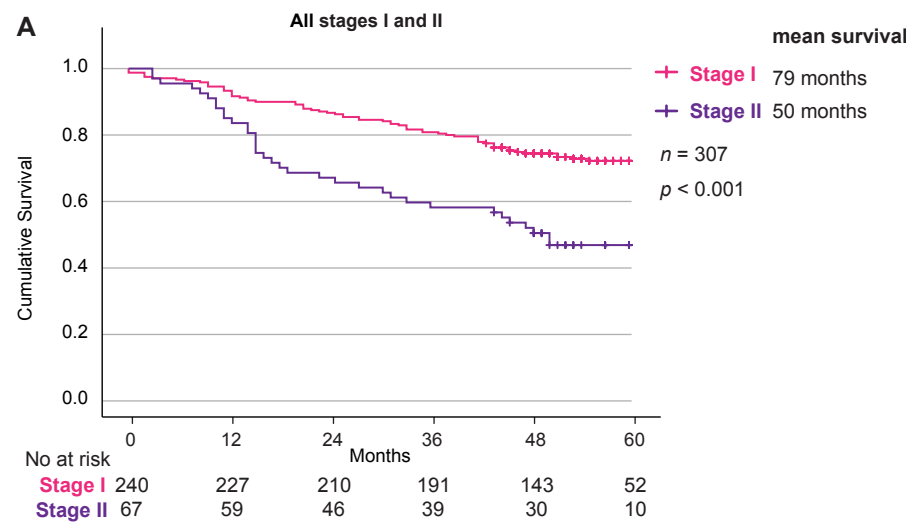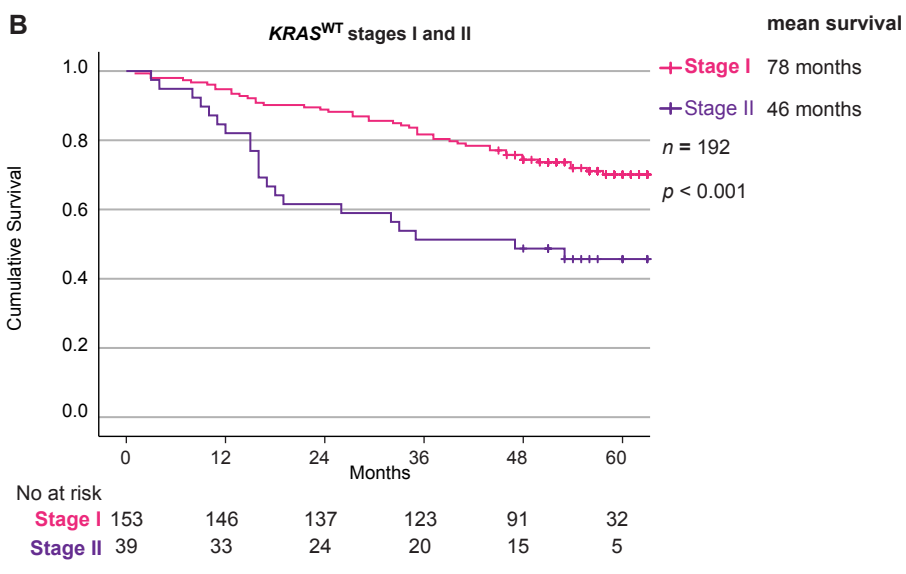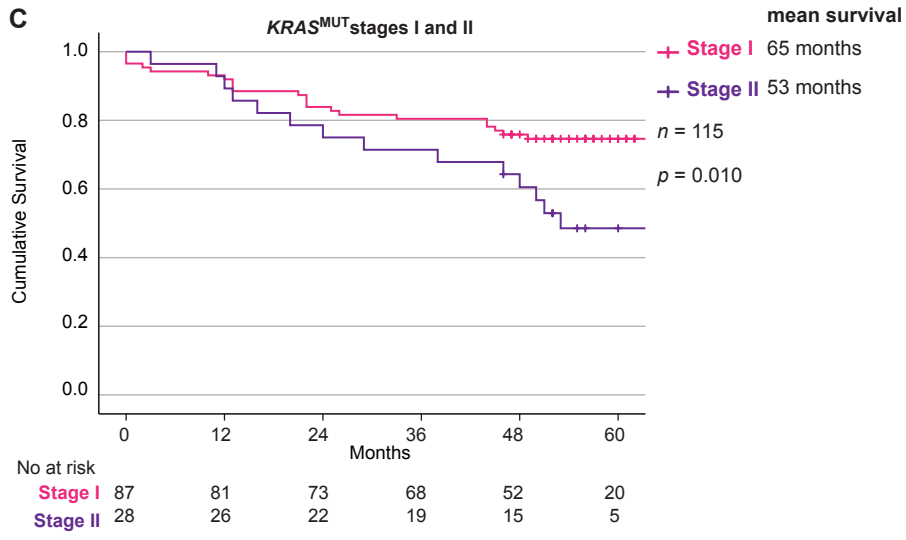

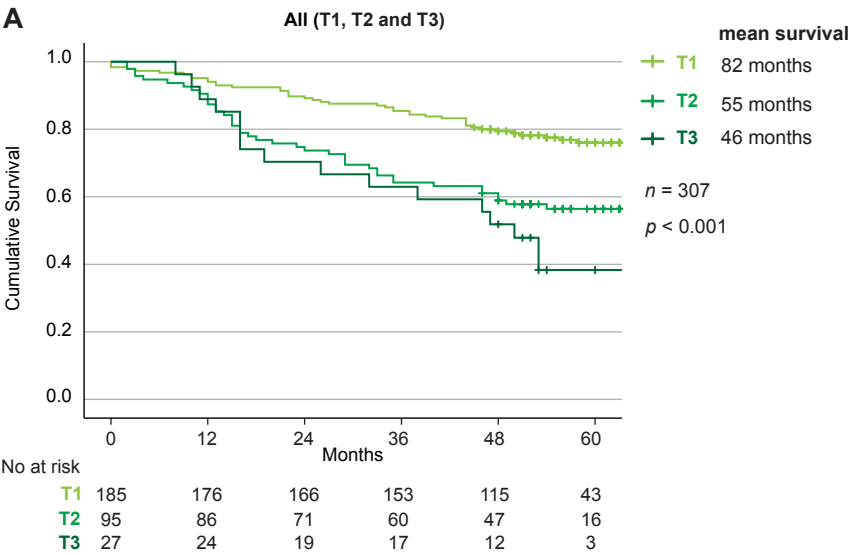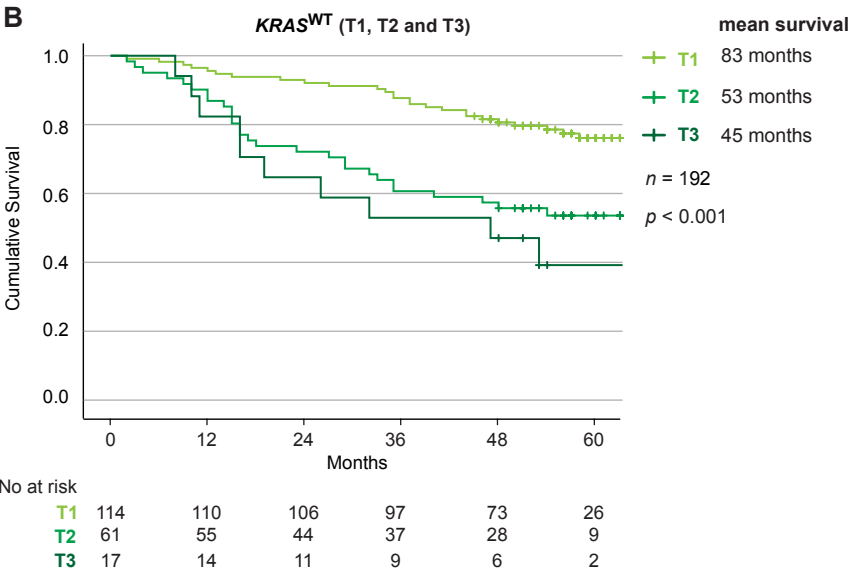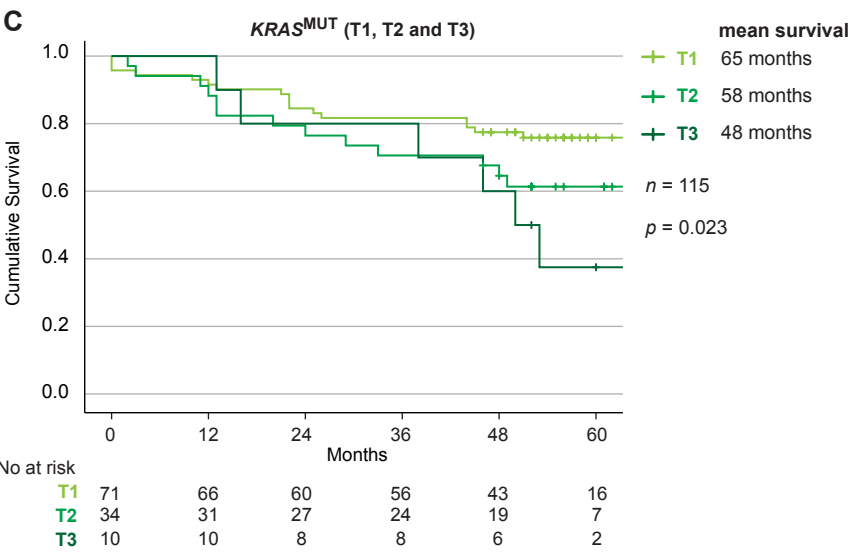

Supplement: Supplementary Figure 1 — Kaplan-Meier estimates of overall survival for resected Stage I-II NSCLC patients stratified by KRAS mutational status. (A) No mutation in KRAS (wildtype, KRAS WT), with all KRAS mutations (KRAS MUT). (B) Only KRAS-G12C mutations (KRAS MUT G12C), KRAS mutations other than G12C (KRAS MUT not G12C). [file DataSheet_1.pdf]
